# A perspective on three sustainable hydrogen production technologies with a focus on technology readiness level, cost of production and life cycle environmental impacts

**DOI:** 10.1016/j.heliyon.2024.e26637

**Published:** 2024-02-22

**Authors:** Yunfei Li, Richen Lin, Richard O'Shea, Vaishali Thaore, David Wall, Jerry D. Murphy

**Affiliations:** a MaREI Centre for Energy Climate and Marine, Environmental Research Institute, University College Cork, Cork, T23 XE10, Ireland; b Key Laboratory of Energy Thermal Conversion and Control of Ministry of Education, School of Energy and Environment, Southeast University, Nanjing, 210096, China; c Civil, Structural and Environmental Engineering, School of Engineering and Architecture, University College Cork, Cork, T12 YN60, Ireland

**Keywords:** Hydrogen economy, Sustainable hydrogen production technologies, System comparison, Technology optimization, Levelized cost of hydrogen, Life cycle environmental impacts

## Abstract

Hydrogen will play an indispensable role as both an energy vector and as a molecule in essential products in the transition to climate neutrality. However, the optimal sustainable hydrogen production system is not definitive due to challenges in energy conversion efficiency, economic cost, and associated marginal abatement cost. This review summarises and contrasts different sustainable hydrogen production technologies including for their development, potential for improvement, barriers to large-scale industrial application, capital and operating cost, and life-cycle environmental impact. Polymer electrolyte membrane water electrolysis technology shows significant potential for large-scale application in the near-term, with a higher technology readiness level (expected to be 9 by 2030) and a levelized cost of hydrogen expected to be 4.15–6 €/kg H_2_ in 2030; this equates to a 50% decrease as compared to 2020. The four-step copper-chlorine (Cu–Cl) water thermochemical cycle can perform better in terms of life cycle environmental impact than the three- and five-step Cu–Cl cycle, however, due to system complexity and high capital expenditure, the thermochemical cycle is more suitable for long-term application should the technology develop. Biological conversion technologies (such as photo/dark fermentation) are at a lower technology readiness level, and the system efficiency of some of these pathways such as biophotolysis is low (less than 10%). Biomass gasification may be a more mature technology than some biological conversion pathways owing to its higher system efficiency (40%–50%). Biological conversion systems also have higher costs and as such require significant development to be comparable to hydrogen produced via electrolysis.

## Highlights

• Polymer Electrolyte Membrane is the most suitable technology for green hydrogen.
• The cost of hydrogen from electrolysis is expected to be less than €6/kg by 2030.
• Nuclear power fuelled water thermochemical cycle may be a route to hydrogen.
• Hydrogen via bio-photolyis can be limited in efficiency to less than 10%.
• Hydrogen via biomass steam gasification has potential efficiencies of c. 50%.

## List of abbreviations including units and nomenclature

ADP-a: Abiotic resource depletion potential
ADP: Adenosine diphosphate
AE: Alkaline electrolysis
AEC: Alkaline electrolysis cell
AP: Acidification potential
ATP: Adenosine triphosphate
CAPEX: Capital expenditures
CCS: Carbon capture and storage
CEPCI: Chemical engineering plant cost index
Cu–Cl: Copper-chlorine cycle
DC: Direct current
e: Electrons
EED: Electro-electrodialysis
EP: Eutrophication potential
EU: European Union
Fdred: Ferredoxin
Fdox: Flavodoxin
GHG: Greenhouse gas
GWP: Global warming potential
H^+^: Hydrogen proton
H_2_: Hydrogen
HHV: High heating value
HI: Hydriodic acid
H_2_O: Water
H_2_SO_4_: Sulfuric acid
HT: High temperature
JAEA: Japan Atomic Energy Agency
JRC: Joint Research Centre
LCA: Life cycle assessment
LCOH: Levelized cost of hydrogen
LHV: Low heating value
LT: Low temperature
MoS_2_: Molybdenum disulfide
ODP: Ozone depletion potential
O&M: Operation and management
OPEX: Operating expenditures
Pd: Palladium
PEM: Polymer electrolyte membrane
PEMEC: Polymer electrolyte membrane electrolysis cell
PIK: Potsdam Institute for Climate Impact Research
Pt: Platinum
PSI: Photosystem
SO_2_: Sulfur dioxide
SOE: Solid oxide electrolysis
SOEC: Solid oxide electrolysis cell
S–I: Sulfur-iodine cycle
TRL: Technology readiness level
Mt/a: Million tonnes per year
V: Voltage
A: Ampere
A cm^−2^: Ampere per square centimetre
m^2^: Square meter
K: Kelvin
€/a: Euro per year
€/kWh: Euro per kilowatt hour
€/kg: Euro per kilogram
Wt: Weight
m^3^H_2_h^−1^: Cubic meter hydrogen per hour

## 1 Introduction

Developing renewable energy systems is strategically imperative for the energy transition. The range of technologies available has different characteristics, advantages, disadvantages, environmental impacts and levels of sustainability. It is essential to optimise the sustainability of such renewable energy technologies by protecting the environment, minimising climate impact, and optimizing sustainable economic and social development. Hydrogen (H_2_) is a versatile energy carrier with the potential to be utilized as an alternative to fossil fuels in a compressed form, such as for use directly in fuel cells and in hard-to-abate sectors such as heavy-duty vehicle transportation.

In the last five years, interest in hydrogen has soared: many organisations, regions, and companies consider hydrogen as indispensable to achieving the Paris Agreement's objective of maintaining global warming below 2 °C and closer to 1.5 °C [1]. The different techniques for sustainable hydrogen production, end-use technologies, and applications define the technological boundaries of the hydrogen economy. Current greenhouse gas (GHG) emissions from the reforming of fossil fuels to produce hydrogen account for 2% of global CO_2_-eq emissions, roughly 900 Mt CO_2_-eq annually [2]. Scaling up the hydrogen economy may be problematic if GHG-emitting technologies continue to be used to produce hydrogen. It is critical that hydrogen should be produced with as low a GHG footprint as feasible and at an attractive cost comparable to fossil-derived hydrogen. To accomplish the decarbonisation of hydrogen production technologies, several obstacles must be overcome, including for electricity sources, sustainable hydrogen infrastructure, and social acceptance.

In the European Union's (EU) hydrogen strategy, the target for sustainable hydrogen production from electrolysis (from a base of 100 Megawatt (MW) in 2021) is 6 Gigawatt (GW) by 2024; this is equivalent to an annual hydrogen yield of c. 1.6 million tonnes (Mt H_2_/a). The target for 2030 is 40 GW, corresponding to 10.6 Mt H_2_/a [3]. However, natural gas steam reforming (grey hydrogen) accounts for 48% of current EU hydrogen production, while oil reforming and coal gasification produce 30% and 18% respectively; sustainable hydrogen only accounts for 4% of the total [[4], [5], [6]]. Even though the current hydrogen output in the EU (from all sources) is approximately 9.8 Mt/a [7], sustainable hydrogen production only accounts for 0.39 Mt/a; this is significantly less than the EU 2030 hydrogen strategy target.

The main sustainable hydrogen production technologies may be sub-divided into three categories whose share of the market may be dictated by specific geopolitical regions: 1. Water electrolysis (such as alkaline, polymer electrolyte membrane, and solid oxide [8]) is likely to be associated with wind and tidal power in temperate oceanic zones (such as west of France, Britain and Ireland) or photovoltaics in sunnier climates (such as sub Saharan Africa) [9]; 2. Water thermochemical cycles (such as copper-chlorine and sulfur-iodine cycle [10]) are more likely to be associated with large nuclear facilities [11]; 3. Biomass-based hydrogen production (such as biomass gasification and biological conversion [12]) is more likely in well forested areas (potentially linked with paper and pulp industries) such as Canada and Scandinavia [13].

Sustainable hydrogen infrastructure must be addressed for large-scale development. For example Mijndert et al. [14] highlighted the lack of widespread availability of low cost renewable electricity, especially in regions where non-renewable electricity production dominates as a major barrier; this is of issue for water electrolysis. For biomass based hydrogen production, microalgae (including cyanobacteria and green algae) break down water molecules into oxygen and hydrogen in the presence of carbon dioxide and light in direct biophotolysis process. Vineet et al. [15] found that the barrier for large-scale application of direct biophotolysis technology lies in low photo-hydrogen energy conversion efficiency (less than 10%) and relatively expensive infrastructure. Thus, it is necessary to compare the technical characteristics, advantages and disadvantages of sustainable hydrogen production technologies to identify the difficulties that need to be overcome to allow for future large-scale application. For other issues such as the public acceptability of the hydrogen economy, it is necessary to address and communicate to society at large the evidence-based research on economic and life cycle environmental impacts of hydrogen production technologies. This information must also be synthesised and used to ascertain the carbon footprint and sustainability of sustainable hydrogen production technologies [16].

Taking account of the different issues such as low levels of sustainable hydrogen production, lack of large-scale infrastructure development, immaturity of some technologies, and safety concerns, many existing studies have investigated optimal sustainable hydrogen production technologies. Ishaq [17] modelled water electrolysis sustainable hydrogen production systems based on geothermal energy and solar PV, and compared the system efficiency of water electrolysis and biomass gasification technologies. The study concluded that biomass gasification had higher exergy (49.8%) and energy efficiency (53.6%) than water electrolysis hydrogen production from geothermal power and solar PV. Ahmad et al. [18] conducted techno-economic assessments of sustainable hydrogen production technologies including; dark fermentation (a biological reaction in which fermentative bacteria convert organic compounds to alcohols, acetone, H_2_, and CO_2_ under anaerobic conditions), photo fermentation, biomass gasification, plasma gasification (a thermal process using plasma to convert biomass into syngas including carbon monoxide and hydrogen) and pyrolysis (thermal decomposition of biomass in the absence of oxygen to biochar, bio-oil and gas). The analysis concluded that dark fermentation performed better from a technical point of view, and the cost of sustainable hydrogen from gasification and fermentation was lower than that from plasma gasification and pyrolysis. Previous studies mainly concentrated on reviewing specific technologies, such as exclusively examining water electrolysis [19] or microbial hydrogen production [20], or limited their comparisons to certain aspects of hydrogen production, such as solely focusing on technical parameters [21] or economic parameters [22]. These studies did not arrive at a definitive answer regarding the optimal hydrogen production technology, taking into account various perspectives such as technical, economic, and environmental aspects.

This study aims to serve as a reference for the large-scale industrial application of hydrogen production technology. To achieve this, technologies attaining a Technology Readiness Level (TRL) between 5 and 9 are encompassed in this paper, signifying their prior validation in practical settings through project-based experimentation. This study aims to provide a comprehensive review of the state-of-the-art of sustainable hydrogen production technologies, including water electrolysis, water thermochemical cycles and biomass-based hydrogen production.

## 2 Sustainable hydrogen production technologies

### 2.1 Hydrogen production from water electrolysis

During electrolysis, water undergoes a decomposition reaction under the influence of direct current and produces oxygen and hydrogen simultaneously (Eq. (1)). As hydrogen and oxygen are generated at the anode and cathode separately, they can be collected and stored easily. Electrolysis techniques can be categorized based on the electrolytes employed: alkaline electrolysis (AE); polymer electrolyte membrane (PEM) electrolysis; solid oxide electrolysis (SOE); and anion exchange membrane (AEM) electrolysis [5]. However, the emerging AEM technology is presently confined to a TRL range between 2 and 3 [23]. The existing research dedicated to AEM systems have primarily concentrated on laboratory-scale investigations, primarily centering on the development of electrocatalysts, membrane materials, and operational mechanisms [24]. Consequently, comprehensive data pertaining to AEM system/system energy efficiency, system construction costs, hydrogen production costs, and environmental impact remain presently unavailable [25].Therefore, the focal point of this paper centres on the remaining three technologies.

(1) $$2H_{2}O\;\to \;2H_{2}+O_{2}$$

#### 2.1.1 Alkaline electrolysis

**Fig. 1:**
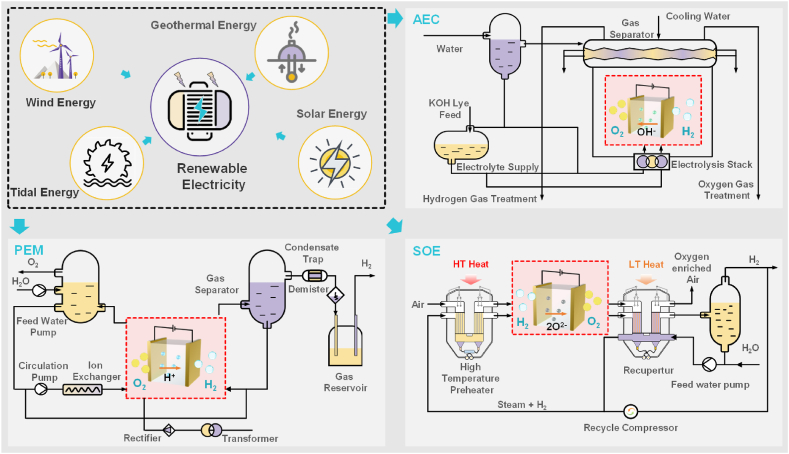
Simplified layout of three water electrolysis systems for sustainable hydrogen production adapted from Refs. [31,32] (AEC: alkaline electrolysis cell; PEM: polymer electrolyte membrane; SOE: solid oxide electrolysis; HT: high temperature; LT: low temperature; KOH: potassium hydroxide).

Since 1920, AE has been in commercial use in the industrial sector. Its advantages include the low capital investment requirement and the low reliance on noble metals and catalysts. Disadvantages include large electricity losses (approximately 40% [26]) and slow start-up speed (which is problematic for association with variable renewable electricity). These disadvantages hinder the large-scale commercial construction and use of AE systems. The typical configuration of AE includes two electrodes (cathode and anode) which are separated by a diaphragm. An hydroxide ion (OH^−^) conducting membrane separates the electrolyte compartments associated with the cathode and anode, allowing for the permeation of OH^−^, but not the gases produced (H_2_ and O_2_), H^+^, electrons, and K^+^/Na^+^ [[27], [28], [29]]. Hydrogen gas is formed on the cathode through proton reduction (Eq. (2)) when electricity is applied to water, while hydroxide ions are oxidized with the production of oxygen as a by-product on the anode (Eq. (3)) [30]. Fig. 1 represents the technical principle and layout of the alkaline electrolysis cell (AEC) system.

(2) $$\text{Cathode}:4H_{2}O+4e^{‐}\;\to \;2H_{2}+4{\text{OH}}^{‐}$$

(3) $$\text{Anode}:4{\text{OH}}^{‐}\;\to \;O_{2}+2H_{2}O+4e^{‐}$$

For large-scale implementation of AE, a potential technological barrier is efficiency, including system efficiency and electrolysis efficiency. System efficiency differs substantially depending on unit sizes as well as other parameters; nevertheless, a large proportion of the difference in literature can be attributed to the boundaries of the system considered. The whole system efficiency (50%–60%) is approximately 10% lower than the electrolysis efficiency (58%–70%) [26]; this can be attributed to current rectification, purification, compression, and storage. Additionally, system efficiency could be further affected by the electrolysis temperature, plant capacity, and system age. For electrolysis efficiency, many internal and external variables can affect both the electrical behaviour and efficiency of electrolysis cells, including the concentration and purity of the electrolyte, the type and shape of the electrodes, and the cell temperature and pressure [6].

Table 1 compares the main characteristics of AE, PEM, and SOE system. The electrolyte is not consumed in the AE process; it only carries the ionic charges required for water decomposition to oxygen and hydrogen. The majority of studies assessed the use of potassium hydroxide (KOH) in traditional electrolysers at concentrations ranging from 25% to 30% by mass [33]. Studies also examined the use of sodium hydroxide (NaOH) and sodium chloride (NaCl) as electrolytes [34]. Hassen et al. [35] found that the KOH electrolyte is more effective than NaOH at the same concentration and under the same temperature, pressure, and voltage conditions, due to the differences in ionic conductivity and purity.

**Table 1:** Comparison of different characteristics of water electrolysis-based hydrogen production technologies.

	AEC^a^	PEM^a^	SOE^a^
TRL^a^	9 [51]	8 [51]	6 [51]
Expected TRL 2050	9 [51]	9 [51]	9 [51]
Typical electrolyte	Aqueous potassium hydroxide (20–40 wt% KOH) [52]	Polymer membrane (e.g. Nafion) [38,39]	Yttria Stabilised Zirconia (YSZ) [53]
Anode	Ni or Ni–Co alloys	RuO_2_ or IrO_2_ [54]	LSM/YSZ [53]
Cathode	Ni or Ni–Mo alloys [38]	Pt or Pt-Pd [54]	Ni/YSZ [53]
Cell voltage (V)	1.8–2.4 [55]	1.8–2.2 [55]	0.7–1.5 [53]
Current density (A cm^−2^)	0.2–0.4 [54]	0.6–2.0 [54]	0.3–2.0 [53]
Cell area (m^2^)	<4 [52]	<0.3 [52]	<0.01 [52]
Voltage efficiency (%)	62-82 [55]	67-82 [55]	77-85 [42]
Operating temperature (◦C)	60-80 [39]	50-80 [55]	650-1000 [56]
Operating pressure (bar)	<30 [52]	30-80 [52]	<25 [52]
Production rate (m^3^H_2_h^−1^)	<760 [52]	<40 [52]	<40 [52]
Stack energy (kWh_el_m^3^H_2_^−1^)	4.2–5.9 [55]	4.2–5.5 [55]	>3.2 [52]
System energy (kWh_el_m^3^H_2_^−1^)	4.5–6.6 [57]	4.2–6.6 [57]	>3.7
Gas purity (%)	>99.5 [58]	99.99	99.9
Cold-start time (min.)	<60 [59]	<20 [59]	<60
System response	Seconds [52]	Milliseconds [52]	Seconds
Stack lifetime (h)	60,000–90,000 [57]	20,000–60,000 [57]	<10,000 [57]
Capital cost per stack 2020 (€_2021_/kW)	1000-1200^d^ [59]	1860-2320^d^ [59]	>2000^d^ [59]
Capital cost per stack 2030 (€_2021_/kW, estimated)	611 [26]	978 [26]	1902 [26]
Stack efficiency (LHV) range 2020 (%)	58–70% [14]	58–65% [14]	81–83% [14]
Stack efficiency (LHV) range 2050 (%, estimated)	61–80% [60]	70–74% [60]	88–90% [60]
Advantages	Long life span	High current density	High system efficiency
	Minimal expense	Compact system layout	Less electricity utilization
	High technology readiness level	Fast response to current change	Expected cost reduction
	Large stack size		Integration with other technologies
Disadvantages	Low current density	Noble metal material requirement	Extraction and utilization of cathodic Lanthanide rare earth elements may cause environmental damage [43]
	Corrosive electrolyte	Short life span	Unstable electrodes
		High membrane expense	Sealing problems
Barriers for large-scale application	Accessibility to low cost and abundant electricity	Accessibility to low cost and abundant electricity	Accessibility to low cost and abundant electricity; immaturity of technology

b: The global share of renewable electricity in total electricity output was approximately 27% at the end of 2019, including 11% produced by wind turbines and solar photovoltaic, which potentially can be used to produce sustainable hydrogen [61].

c: Adequate renewable electricity for large-scale deployment of electrolysis is assumed to be available based on the existing net-zero commitments [62,63].

a AEC: alkaline electrolysis cell; GHG: greenhouse gas; LHV: Low heating value; LSM: La_0.8_Sr_0.2_MnO_3_; PEM: polymer electrolyte membrane; SOE: solid oxide electrolysis; TRL: technology readiness level; wt: weight.

d Updated capital cost according to Chemical Engineering Plant Cost Index (CEPCI). CEPCI_2020_ = 596.2; CEPCI_2021_ = 708.0. Calculation formula: cost at 2021 = cost at 2020 · $\frac{CEPCI\;index\;at\;2021\;}{CEPCI\;index\;at\;2020}$ [64]).

With regards to electrodes, Slama et al. [36] investigated different types of materials, such as stainless steel, copper, aluminium, bronze, graphite, and lead; the study found stainless steel to be an optimal choice due to its excellent corrosion resistance, low price and electrolytic performance. Despite the high TRL of AE, it presents the disadvantages of low current density and the use of a corrosive electrolyte; this issue should be addressed for more economic and sustainable operation. Of issue when considering electrolysis associated with wind turbines or other variable renewable electricity generators is the cold start time, which is of the order of 60 min.

#### 2.1.2 Polymer electrolyte membrane electrolysis

General Electric pioneered the PEM electrolysis technology in the 1960s. PEM is less established than AE systems and is primarily employed for small-scale applications [37]. The primary advantages of the PEM technology are associated with the high electricity-to-hydrogen conversion efficiency (approximately 60%), the production of high purity hydrogen (99.99%), as well as the ability to operate flexibly [38]; this is of huge importance for integration with variable renewable electricity generators such as solar and wind. The disadvantages currently include high catalyst (such as Platinum and Palladium) and membrane material expenses, the system's complexity owing to high-pressure operation, strict water purity standards, and the system's shorter lifetime when compared to AE systems [39].

The principle of PEM operation is as follows: a polymer membrane with high proton conductivity is used instead of an aqueous electrolyte in this electrolysis cell. Only protons and electrons can be transferred between the electrodes. During the electrolysis process, H_2_ is generated in the cathode layer, while O_2_ is produced in the anode layer. Fig. 1 represents the technical principle and layout of PEM electrolysis system, the electrode reactions are detailed in Eq. (4) and Eq. (5):

(4) $$\text{Cathode}:2H^{+}+2e^{‐}\;\to \;H_{2}$$

(5) $$\text{Anode}:H_{2}O\;\to \;1/2O_{2}+2H^{+}+2e^{‐}$$

For the AE water electrolysis process, hydrogen and oxygen are electrochemically produced from water at the cathode and anode electrodes respectively. In contract to this, water is pumped to the anode in PEM water electrolysis, where it is split into O_2_, protons (H^+^), and electrons (e^−^). The proton conducting membrane transports these protons to the cathode side [40]. The electrons leave the anode by way of the external electricity circuit, which supplies the reaction's driving force in the form of a cell voltage. The protons and electrons recombine to produce hydrogen at the cathode, hence the half-reaction equations for the two technologies differ. PEM electrolysis can produce a purer form of hydrogen (99.99%), whereas the purity of hydrogen produced by AE is 99.5% [41].

The basic technical parameter comparison between PEM and the other two electrolysis systems (AE and SOE) is summarised in Table 1. The PEM is the most adaptable of the three electrolytic systems because it can accommodate rapid ramp up and down as well as intermittent loads, while the AE can only accommodate moderate ramping [40]. The SOE can only operate well under stable conditions [42]. Furthermore, membrane material degradation [43]during the electrolysis process shortens the lifespan of PEM equipment, giving AE the edge in terms of cost-effectiveness and adaptability [44].

Current research directions mainly focus on developing suitable electrode materials and structures such as electrode layers, polymer membranes, and catalysts to reduce infrastructure construction and operating costs; this should make PEM electrolysis technology more cost competitive in the industry sector. Molybdenum disulfide (MoS_2_) and related substances have good catalytic activity [45], and according to the findings of Mo et al. [46], the addition of first-row transition metal elements to MoS_2_ can enhance the catalytic activity of monolayer MoS_2_, particularly Co-^s^MoS_2_, which is capable of competing with Pt-based catalysts in industry, and as such is an efficient alternative catalyst material.

#### 2.1.3 Solid oxide electrolysis

In contrast to AE and PEM, superheated steam is used as a feedstock in the SOE technology; a ceramic membrane is utilized as the electrolyte to conduct O^2−^ ions at elevated temperatures. At temperatures ranging from 923 K to 1273 K, SOE cells typically operate at current densities of more than 1.0 A/cm^2^ and a single cell voltage of roughly 1.3 V [47]. As a result of the presence of superheated stream, this method consumes significantly less electrical energy than other water electrolysis technologies, allowing for electrical energy savings. The oxygen-ion conducting membrane is required for the electrolysis process to take place. The technological concept and process of SOE system is depicted in Fig. 1, and the electrode reactions are detailed in Eq. (6) and Eq. (7):

(6) $$\text{Cathode}:2H_{2}O+4e^{‐}\;\to \;2H_{2}+2O^{2‐}$$

(7) $$\text{Anode}:2O^{2‐}\;\to \;O_{2}+4e^{‐}$$

The electrolyser lifespan is currently a key barrier to large scale SOE commercialization, because the high operating temperature has a detrimental effect on SOE durability. The yearly degradation rate required for a SOE cell to achieve economically viable status in comparison to low temperature electrolysis has been assessed at 8%, in contrast to the currently observed degradation rate of 17% [48]. The system's efficiency can be irreparably reduced because of heating and cooling which can create tiny cracks on the membrane surface. Thus, a primary objective of on-going research is to identify electrolyser materials that are sufficiently robust when exposed to high temperatures and humidity to ensure long-term performance stability. Hauch et al. [49] discovered that strontium-doped lanthanum manganite may be an excellent anode material due to its stability in thousands of hours of testing; this can be attributed to its porous microstructure. Ni/YSZ has been utilized for over three decades and therefore is still a typical choice for the cathode material. However, as demonstrated by the rapid drop in initial conductivity, Simwonis et al. [50] found that the agglomeration of nickel particles casts doubts on the material's stability. Several additional alternative cathode materials have since been developed, including lanthanide metal-based and titanate-based composites [49].

### 2.2 Hydrogen production from water thermochemical cycles

Similar to electrolysis, water can be decomposed into oxygen and hydrogen in thermochemical pyrolysis processes. The term "water thermolysis" refers to the thermal breakdown reaction that occurs in a single step [65]. The reaction system should operate at a reasonably high temperature (greater than 4273 K) [66] because one-step thermolysis requires a considerable amount of heat energy. This high temperature requirement poses a great challenge for large scale industrial utilization. Another challenge is that water thermolysis produces a mixture of hydrogen and oxygen that is easily recombined back into water, which is difficult to sequentially segregate. As a result, one-step water thermolysis is not in commercial or industrial use currently.

To overcome this limitation a lot of research has focused on water thermochemical cycles as a solution to this problem. In these cycles, water molecules first react with supplementary chemicals (such as sulfur – iodine and copper chlorine) to produce intermediary compounds, which then release hydrogen and oxygen. These cycles, which involve several supplementary and intermediary processes, only accept water as a feedstock, and the final products are oxygen and hydrogen, with the supplementary chemicals left in the system for the next cycle [67]. This greatly enhances potential for commercial and industrial application. Thermochemical hydrogen production may be subdivided into three categories: multi-step cycles involving sulfur-iodine (S–I: section 2.2.1) or copper-chlorine (Cu–Cl: section 2.2.2), and two-step cycles using metal oxides (M*O*_*x*_) [11].

#### 2.2.1 Sulfur-iodine cycle

**Fig. 2:**
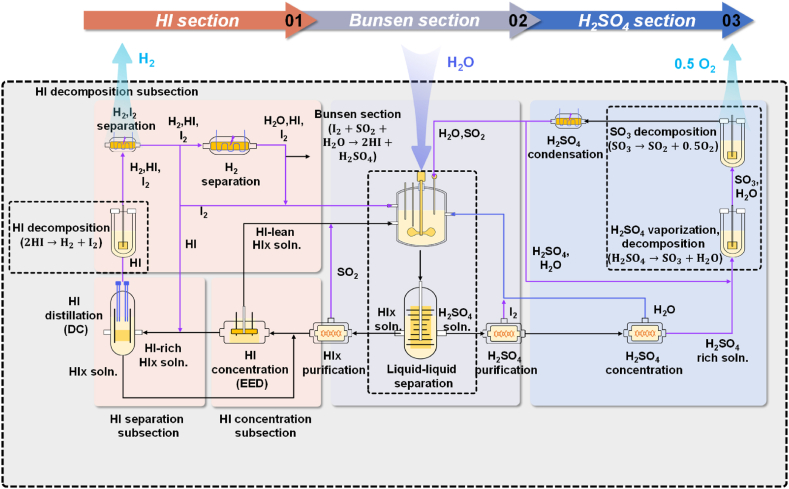
Schematic of the sulfur-iodine cycle for sustainable hydrogen production [79] (DC: direct current; EED: electro-electrodialysis; soln.: solution; HI: hydriodic acid; I_2_: iodine; H_2_SO_4_: sulfuric acid; Hix: HI–H_2_O–I_2_; SO_2_: sulfur dioxide; Soln: solution).

High temperature heat (above 1200 K) from nuclear reactors was considered a viable energy source for production of transportation fuels such as ammonia (NH_3_) and methanol (CH_3_OH) after the oil crises of the 1970s; the chemical reactions to break water molecules into oxygen and hydrogen may be optimised to this nuclear heat source [68]. As a result, by the mid-1970s, General Atomics had introduced and developed the sulfur-iodine cycle (S–I) in the United States [69]; this was followed by the European Joint Research Centre (JRC) in Ispra, Italy, the Japan Atomic Energy Agency (JAEA) [70], and further work in Italy [71]. The S–I pilot plant at JAEA in Japan has demonstrated 30 L/h of hydrogen production [70]; France, Canada, China, and Korea started nuclear hydrogen manufacturing initiatives in the 2020s [[72], [73], [74]]. Solar energy can serve as a viable heat source for the S–I cycle [75]. Table 2 depicts the three basic chemical processes (Bunsen reaction, HI decomposition, and sulfuric acid decomposition) in the S–I cycle. When liquid water is added to a system containing gaseous SO_2_ and solid I_2_ at a temperature in the range between 293 and 393K, an exothermic Bunsen reaction occurs, resulting in the formation of two acids: H_2_SO_4_ (sulfuric acid) and HI (hydroiodic acid), which are immiscible aqueous concentrated acids. The decomposition of HI is particularly energy expensive and therefore requires a high temperature (Table 2), which has a detrimental impact on the cycle's overall efficiency [76]. One of the key challenges of the S–I cycle is to eliminate water and iodine surpluses, or, to develop separation technologies that require less energy than distillation. In on-going research, the TRL of the S–I cycle is stated as 6 [77], which is lower than the aforementioned water electrolysis hydrogen production technologies (PEM and AE). Fig. 2 depicts the basic concept of the S–I cycle.

**Table 2:** Characteristics of sulfur-iodine water thermochemical cycle hydrogen production technology [78].

Reaction name	Reactions	Temperature (K)	ΔH (kJ/mol)
Bunsen reaction	I_2_ + SO_2_ + 2H_2_O → H_2_SO_4_ + 2HI	293–393	−75 ± 15
HI decomposition	2HI → H_2_ + I_2_	1073–1273	186 ± 3
Sulfuric acid decomposition	H_2_SO_4_→ SO_2_ + 1/2O_2_ + H_2_O	573–773	12

A key barrier to the large-scale application of the S–I cycle mainly lies in the optimization of the reaction process, which should consider the following three aspects: ***(1) Improvement of the operating conditions for high-efficiency Bunsen reaction such that more HI and H***_***2***_***SO***_***4***_ ***can be produced***. Lee et al. [80] stated that the ideal operating parameters for the Bunsen reaction is 11 mol of excess water and 4 mol of excess iodine at 330 K, whereas the permissible window for the process reaction is between 11 and 13 mol of excess water, and 4–6 mol of excess iodine, at a temperature of 330–350 K. This condition favours obtaining an HI concentration that is over-azeotropic while avoiding iodine solidification and side reactions. Nafees et al. [81] suggested that it is preferable to operate the reactor at roughly 333K, 4 bar(g), with feed concentration ratios of HI/I_2_/H_2_O of 1/2.8/7.7. ***(2) Separation of HI and H***_***2***_***SO***_***4***_ ***and subsequent purification following the Bunsen reaction.*** Bai et al. [82] investigated the reverse Bunsen reaction kinetics to determine the reaction mechanism between H_2_SO_4_ and HI. Their findings revealed that reaction temperature had the greatest impact on the purification of the H_2_SO_4_ phase, with 99% of contaminants eliminated at temperatures higher than 403 K using N_2_ stripping. The iodine concentration was crucial in determining the reactions during the purification of the HI_x_ phase. By raising temperature, the flow rate of stripping gas, and the concentration of iodine, the purifying effect can be enhanced, and side reactions can be effectively inhibited [82]. ***(3) Improvement of the HI decomposition to H***_***2***_ ***and I***_***2***_**.** HI can be decomposed by catalyst to form hydrogen and iodine. Chaubey et al. [83] stated that currently available catalysts include Pt (Platinum)-metal alloys, Pt loaded on supporters, and metal oxide. When it comes to the metals that can be introduced to produce binary catalysts with Pt, Pd (Palladium), Ir (Iridium), Ni (Nickel) and Rh (Rhodium) are frequently mentioned and used. For the supporters of Pt, active carbon, carbon nanotubes, carbon molecular sieve, γ-Al_2_O_3_ and graphite are all frequently utilized materials for the catalysts; Pt/carbon nanotubes have optimum activity and stability in the temperature range 673–873 K. In addition to catalysis, HI can also be decomposed by electrolysis.

#### 2.2.2 Copper–chlorine cycle

The copper-chlorine (Cu–Cl) cycle was established in 1984 and has the advantage of a lower temperature requirement (about 803 K) than S–I cycle [84]. As a result, its operation and material costs are low, allowing for effective integration with different energy systems, particularly solar and nuclear power plants [11]. The Cu–Cl cycle is a hybrid cycle (using both electrical and thermal energy); it consists of three chemical reactions: electrolysis of aqueous CuCl (cuprous chloride) and HCl (hydrochloric acid) to produce hydrogen and CuCl_2_ (copper dichloride), hydrolysis of CuCl_2_ with steam to produce Cu_2_OCl_2_ (melanothallite), and thermolysis of Cu_2_OCl_2_ to produce oxygen [85]. Table 3 outlines the principles of the Cu–Cl cycle.

**Table 3:** Reaction characteristics of Cu–Cl cycle [11,85].

Reaction name	Reactions	Temperature (K)
Electrolysis of CuCl and HCl	2CuCl(aq) + 2HCl(aq) → H_2_(g) + 2CuCl_2_(aq)	298
Hydrolysis of CuCl_2_	2CuCl_2_(s) + H_2_O(g) → Cu_2_OCl_2_(s) + 2HCl(g)	298–648
Thermolysis of Cu_2_OCl_2_	Cu_2_Ocl_2_(s) → 2CuCl(l) + ½ O_2_(g)	648–803

(aq: aqueous; g: gaseous; s: solid; l: liquid; while aqueous solution appears in liquid form, the liquid subscript represents a molten state of the salt instead of aqueous; drying process: CuCl_2_(aq) → CuCl_2_(s) T = 308–353 K.).

The Cu–Cl cycle has a low rate of undesirable reactions and produces no greenhouse gases or other pollutants; however, the Cu–Cl cycle technology lacks technological maturity and has a high cost of equipment, which at this TRL precludes large-scale commercial deployment [86]. According to the number of reactions, Cu–Cl cycles can be subdivided into three-step, four-step, or five-step cycles [87]. Different Cu–Cl thermochemical cycles generate hydrogen energy in distinct ways, with distinct heat and mass transfer mechanisms, distinct intermediate products, and ultimately distinct hydrogen yields. Orhan et al. [88] proposed that one disadvantage of the five-step Cu–Cl cycle is the creation of copper chloride (CuCl) and solid copper which increases the number of solid particles transported and handled inside the cycle. Additionally, estimating the mass and heat transfer mechanisms of solid-fluid or solid-solid mixtures becomes increasingly complicated as a result of incomplete reactions, undesirable by-products, and a resulting drop in overall cycle efficiency [88]. This drawback can be overcome through lowering the number of major reactions in the five-step Cu–Cl cycle and so minimising the creation of unwanted solid particles. Additional benefits of reducing steps in the Cu–Cl cycle include improved reaction kinetics, as well as optimised management of gaseous liquids when compared with solid phase components.

However, shortening the Cu–Cl cycle steps may result in additional issues such as increased heat requirement, increased generation of undesired by-products, and decreased output of the desired products. The requirement for a higher-grade source of heat complicates material selection for constructing and developing the Cu–Cl cycle reactors from a practical engineering viewpoint. Using a life cycle assessment model, Ahmet et al. [89] assessed several water thermochemical splitting cycles and determined that the four-step Cu–Cl cycle had less environmental impact than the three- and five-step cycles.

#### 2.2.3 Two-step thermochemical hydrogen production cycles

As observed in Eqs. (8), (9), a metal oxide serves as an intermediary medium for the breakdown of water during a two-step thermochemical cycle process. Currently available metals are divided into two general categories: volatile metals such as Zn (Zinc) and Sn (Tin), and non-volatile metals such as Fe (Iron) and Se (Selenium) [90]. The advantages of the two-step thermochemical cycle include the production of oxygen and hydrogen in distinct stages, which eliminates the need to separate them. The ability to use high-temperature heat (1780–2600 K [91]) directly can improve system energy efficiency and decreases the requirement for further power generation procedures. There are still drawbacks (such as the difficulty of selection and implementation of high temperature resistant materials) which place tremendous strain on industrial infrastructure when combined with renewable energy utilization [91].

The thermal reduction step is described in Eq. (8):

(8) $$M\;O_{x}\;\to \;M\;O_{x-\delta }+\frac{\delta }{2}\;O_{2}$$

The water splitting step is described in Eq. (9):

(9) $$M\;O_{x-\delta }+\delta \;H_{2}O\;\to \;M\;O_{x}+\delta \;H_{2}$$

Where M are specific metals, such as Zn, Sn, Ce, or Fe. Table 4 depicts recent advances in selected two-step thermochemical cycles. Current research activities on two-step thermochemical cycles can be separated into three distinct phases. The initial phase is identifying, testing, and developing appropriate metal oxide materials (M*Ox* in Eqs. (8), (9))). The second phase involves the examination of two-step cycles at a laboratory scale. The third phase involves the implementation of pilot testing and optimization of the system. At temperatures between 1573 and 1773 K, Antoine et al. [92] observed that doped-ceria and spinel ferrite were the most suitable materials which exhibited strong hydrolysis abilities. Apart from these two materials, no material could produce hydrogen efficiently below 1373 K in literature on the thermochemical hydrogen production cycles. Non-volatile non-stoichiometric oxides (δ in M $O_{x-\delta }$ is not an integer but a decimal number) such as zirconium oxide have provided a new route for the development of thermochemical materials for renewable energy production due to their superior thermo-kinetic characteristics for hydrogen production, good structural stability, and moderate reduction temperature [93]. Heat recovery is crucial to improve the energy conversion efficiency. The inclusion of an oxygen exchange membrane provides the potential for inert gas recovery, and alterations of the catalyst structure should also be taken into account [94]. Solar thermochemical hydrogen production at 1673 K has been proven at a pilot scale, according to Abanades et al. [95]; this establishes a significant benchmark for future development.

**Table 4:** Recent advances in selected two-step thermochemical cycles.

Redox pairs	Thermo reduction step temperature	Efficiency (LHV)		Characteristics	Reactions	Recent advances
		No heat recovery	With heat recovery (50%)			
SnO_2_/SnO	1780K [96]	36.26% [96]	49.61% [96]	1. Volatile cycle with high reduction temperature requirement.	1. SnO_2_ (s) → SnO (g) + 0.5 O_2_ (g) 2. SnO (s) + H_2_O (g)→ SnO_2_ (s) + H_2_ (g)	As a volatile metal, SnO_2_/SnO is often compared with ZnO/Zn in hydrogen production rate, hydrolysis speed, activation energy, reaction orders, and kinetic rate laws [97]. Meanwhile, thermochemical analysis and solar-to-fuel energy conversion efficiency were evaluated by chemistry software and a database [96].
				2. High theoretical energy conversion efficiency.		
				3. Material loss during reduction process.		
				4. High SnO hydrolysis rate (98%).		
				5. Disproportionation reaction at temperatures above 600 °C: SnO → SnO_2_ + Sn.		
ZnO/Zn	2300K [98]	53.2% [98]	–	1.Volatile cycle with higher reduction temperature requirement than SnO_2_/SnO.	1. ZnO (s) → Zn (g) + 0.5 O_2_ (g) 2. Zn (s) + H_2_O (g)→ ZnO (s) + H_2_ (g)	The research directions of ZnO/Zn in recent years mainly focus on: 1. heat transfer mechanism analysis [99]. 2. thermodynamic efficiency evaluation and promotion pathways [100]. 3.Reactor construction [101,102].
	2000K [103]	14.0% [103]	>30% [103]	2. Fast hydrolysis rate.		
	2300K [104]	29% [104]	–	3. Insufficient hydrolysis due to ZnO deposition on the surface of Zn.		
				4. Material loss during reduction process.		
Fe_3_O_4_/FeO	1875K [105]	20.4–25.1% [105]	50.7–62.5% [105]	1. High theoretical energy conversion efficiency.	1. Fe_3_O_4_ (s) → 3FeO (s) + 0.5 O_2_ (g) 2. 3FeO (s) + H_2_O (g)→ Fe_3_O_4_ (s) + H_2_ (g)	Recent research mainly focuses on assessing the effect of operation conditions such as temperature, pressure, and steam to feed ratio on the reaction products and conversion rates [106,107].
				2. Materials are more likely to sinter and deactivate when the temperature is higher than 2273 K.		
				3. Non-stoichiometric Fe_1-y_O is present in the reduction products, which is more active and can hydrolyse quicker.		
				4. The reduction temperature requirement is lowered by Fe_3_O_4_ supported on *m*-ZrO_2_ or YSZ.		
CeO_2_/Ce $O_{2-\delta }$	1873K [108]	0.7–0.8% [108]	–	1. Reduction temperature requirement is lower than ZnO/Zn.	1. CeO_2_ → Ce $O_{2-\delta }$ + $\frac{\delta }{2}$ O_2_ 2. Ce $O_{2-\delta }$ + $\frac{\delta }{2}$ H_2_O (g)→ CeO_2_ + δ H_2_	Since this cycle is still in the laboratory stage, the current research focuses on the study of reaction kinetics and the determination of the optimal reaction conditions [109,110].
	1723–1773K [111]	5.25% [111]	–	2. Stable circulation.		
	1800 K [112]	20.2% [112]	29.5% [112]	3. Doping ZrO_2_ can increase reduction rate and reduce temperature requirement.		
	2300–2600K [113]	23–29% [113]	–	4. Non-stoichiometric Ce $O_{2-\delta }$ can persist steadily in air with high activity		

### 2.3 Hydrogen production from biomass

Biomass can be derived from a variety of sources, including grasses, wood, crop residues, agricultural products, animal and plant wastes, food scraps, municipal wastes and algae, and is viewed as a viable substitute for fossil fuels [114]. Direct hydrogen production from biomass can be achieved in two ways according to the mechanism of gas generation: thermochemical processes (including gasification, pyrolysis, and liquefaction techniques [66]) or biological processes (including dark/photo fermentation and bio-photolysis).

#### 2.3.1 Biomass gasification

Biomass gasification is partial oxidation of biomass compounds in presence of air, oxygen or steam to produce gases mainly including of CO, CO_2_, CH_4_ and H_2._ Methane and other hydrocarbons such as tars and char are also produced. In comparison to other waste processing techniques such as landfilling and incineration, biomass gasification has a higher potential for application because it can accept a wide range of inputs, including for diverse feedstocks such as wood and algae, and produce multiple useful products, including hydrogen and carbon monoxide [115].

Drying the feedstock is the first step in the complex process of biomass gasification, which also involves pyrolysis, partial combustion of intermediates, and gasification of the final products. The process is carried out inside a gasifier with the presence of gasifying media which can be air, steam (H_2_O), oxygen (O_2_), or carbon dioxide (CO_2_) [116]; the gasifying media has a significant impact on the product gas calorific value. The heating value can be in the range 4–7 MJ Nm^−3^ for the product gas from air gasification (due to the presence of nitrogen in air), whereas it can rise up to the range of 12–28 MJ Nm^−3^ when oxygen is used as gasifying media (excluding nitrogen in the producer gas) [15].

By lowering the carbon-to-hydrogen (C/H) mass ratio, biomass gasification enhances the product's calorific content due to an increased H_2_ fraction. The gasifying media is essential for turning heavy hydrocarbons and solid char into low-molecular-weight gases like hydrogen and carbon monoxide. The gasifying media, feedstock material, reactor design, reactor temperature and pressure, and catalyst type all play significant roles in the quality of product gas [117]. The two commonly used thermochemical biomass-to-hydrogen technologies are steam gasification and supercritical water gasification with comparisons detailed in Table 5.

**Table 5:** Characteristic comparison of steam gasification and supercritical water gasification technologies.

Technology	Steam gasification	Supercritical water gasification
Reaction process	C + H_2_O → H_2_ + CO CO + H_2_O → H_2_ + CO_2_ CH_4_ + H_2_O → 3H_2_ + CO C_a_H_b_ + aH_2_O → (a + b)H_2_ + aCO [118]	CH_n_O_m_ + (1-m)H_2_O → (n/2+1-m)H_2_ + CO CO + H_2_O → H_2_ + CO_2_ CO + 3H_2_ → CH_4_ + H_2_O CO_2_ + 4H_2_ → CH_4_ + 2H_2_O [119]
Product gas heating value, MJ/Nm^−3^	High 15–20 [120]	High 15–20 [120]
Average H_2_ production (wt%, g H_2_/100 g	Without catalyst: 4g With catalyst: 7g [115]	Without catalyst: 3g With catalyst: 5g [115]
Typical biomass	Lignocellulose, algae, wood saw dust, waste wood, paper, coffee husk, almond shell [117]	Sewage sludge, aqueous sludge, contaminated wastewater, coal wastewater, chicken manure [117]
Reactor	Fluidized bed; upper/lower ventilation gasifier [115]	Continuous reactor; batch reactor [115]
Reactor temperature (K)	973-1473 [117]	663-973 [117]
Catalyst	Dolomite, Ni based catalyst, alkaline metal, alumina, K_2_CO_3_, Na_2_CO_3_, ZnCl [121]	K_2_CO_3_, Na_2_CO_3_, KOH, NaOH, ZrO_2_, Ni/ZrO_2_ [121]
Influential operating parameters	Biomass characteristics, temperature, steam-to-biomass ratio [122]	Temperature, operating pressure, reactant concentration, reaction time [122]
System energy efficiency (LHV)	40–50% [122]	40–50% [122]
Technology readiness level	8 [123]	8 [123]
Advantages	Potential for large-scale industrial production because of minimal ash production and high gasification rate.	High gasification rate without the generation of tar, coke, or secondary pollution.
Disadvantages	Difficult to separate and purify the gas products.	Strict operating conditions and difficult alkaline catalysts recycling process.
Challenges	Reduce tar concentration; develop appropriate catalyst; improve technology readiness level and reduce technology construction and operating costs.	Improve technology readiness level and reduce technology construction and operating costs.

(LHV: low heating value; wt: weight).

For both steam gasification and supercritical water gasification, the introduction of a catalyst can reduce the temperature requirement of the reaction, and promote condensable fraction reforming and tar cracking. Tar is a substantial issue in the biomass gasification process as it can clog equipment (heat exchangers), raise maintenance costs, and complicate overall operation [124,125]). To enhance hydrogen production and carbon conversion efficiency, more recent research has focused on the design and selection of appropriate catalysts. According to Okolie et al. [126], typical alkaline metal catalysts can accelerate the steam gasification and supercritical water gasification process effectively, however, there are limitations such as the difficulty of catalyst recovery, significant loading on the catalyst, and blockages. Chan et al. [127] stated that noble metals, notably Rh (Rhodium) and Ru (Ruthenium), exhibit good catalytic activity in both pathways but cannot be employed widely due to cost constraints. Gai et al. [128] proposed that Ni (nickel)-based catalysts have been commonly utilized as effective catalysts and that it is preferable to employ them in conjunction with other metals. Because of the high solubility in water at high temperatures, the frequently employed Al (Aluminium)-based catalysts for steam gasification are not optimal for supercritical water gasification. According to Gholkar et al. [129], not only do metal oxides possess catalytic activity, but they may also be effective supporters for external metal catalysts: a metal oxide supporter can increase the stability of Ni-based catalysts.

To enhance system operation efficiency and hydrogen production efficiency, much research on biomass gasification has focused on optimizing operating parameters, such as (1) selection of the type, quality and moisture content of the biomass feedstock; (2) developing appropriate density and particle size of the feedstock; (3) investigating steam-to-biomass ratios; (4) finding the applicable air equivalence ratio (the proportion of actual air supplied as compared to the stoichiometric air required for the operation). Schuster et al. [15] claimed that more gaseous products can be produced during gasification by appropriately increasing the ratio of cellulose and hemicellulose to lignin in biomass. Despite the fact that reducing particle size enhances syngas efficiency and decreases tar yields, the particle size should not be decreased below the minimum required level since particle size reduction requires a substantial amount of extra energy input. According to Nader et al. [130], a rise in temperature increases the heating rate of the feedstock particles by producing a wider temperature difference, thus promoting an increase in the reaction rate. Jun et al. [131] found that an increase in air equivalence ratio decreased H_2_ and CO yield whilst increasing CO_2_ concentration, consequently reducing the calorific value of the generated gas.

#### 2.3.2 Biological conversion

**Fig. 3:**
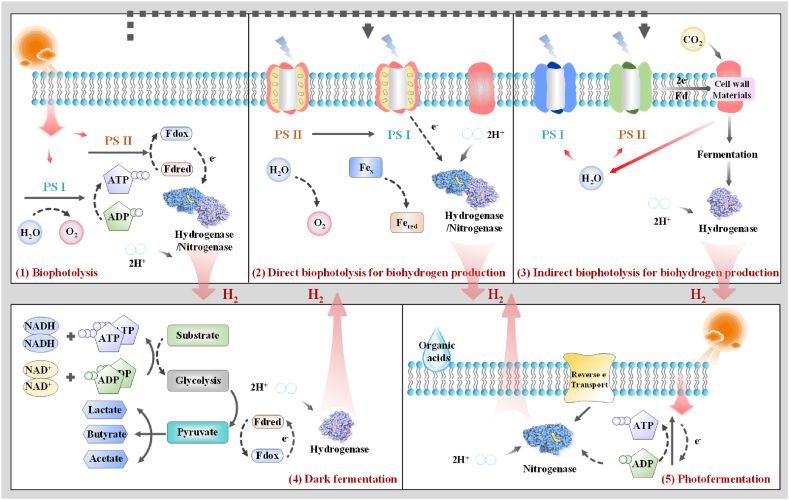
Principle of biological hydrogen production technologies. (1) biophotolysis, (2) direct biophotolysis for biohydrogen production, (3) indirect biophotolysis for biohydrogen production, (4) dark fermentation, (5) photofermentation [144] (ATP: adenosine triphosphate; ADP: adenosine diphosphate; Fdred: ferredoxin; Fdox: flavodoxin; PSI: photosystem I; PSII: photosystem II; NADH: nicotinamide adenine dinucleotide hydrogen).

Biological hydrogen production includes two typical processes: bio-photolysis and fermentative biohydrogen generation [132]. Certain microbes are capable of splitting water and producing H_2_ under light-driven circumstances during the bio-photolysis process; this process can further be classified as direct or indirect bio-photolysis with green algae and cyanobacteria as representative microorganisms. Fermentation (including dark and photo fermentation) is a biological reaction in which microbes convert organic compounds (such as starch and cellulose) to alcohols, acetone, H_2_, and CO_2_ under aerobic or anaerobic conditions. The TRL for both direct and indirect biophotolysis is 4 [133], whereas for photofermentation is 4 [134], and dark fermentation is 7 [135]. Fig. 3 depicts the principle of biological hydrogen production technologies, and Table 6 presents a comparison of the different ways for biohydrogen production.

**Table 6:** Technological characteristics of different biohydrogen production process.

	Direct biophotolysis [145,146]	Indirect biophotolysis [147,148]	Photo fermentation [149,150]	Dark fermentation [[151], [152], [153]]
Substrate	H_2_O	H_2_O, CO_2_	⁃ Simple sugars ⁃ Organic wastes	⁃ Simple sugars ⁃ Organic wastes
Microorganism	Green algae, cyanobacteria	Cyanobacteria	Photoheterotrophic bacteria (*Rhodobacter, Rhodobium, Rhodopseudomonas,* and *Rhodospirillum* strains)	Obligate or facultative anaerobe fermentative bacteria (*Alcaligenes, Bacillus, Clostridia, Citrobacter, Enterobacter, Escherichia coli*)
Reaction	2H_2_O + light →2H_2_+O_2_	1: 6CO_2_ + 6H_2_O → C_6_H_12_O_6_ + 6O_2_ 2: C_6_H_12_O_6_ + 6H_2_O → 12H_2_ + 6CO_2_	CH_3_COOH+2H_2_O + light → 4H_2_+2CO_2_	*Acetic Acid Pathway:* C_6_H_12_O_6_ + 2H_2_O → 4H_2_+2CO_2_+2CH_3_COOH *Butyric Acid Pathway*: C_6_H_12_O_6_ + 2H_2_O →C_4_H_8_O_2_ + 2CO_2_ + 4H_2_
Light/energy requirement	Yes	Yes	Yes	No
By-products	O_2_	CO_2_, O_2_, metabolites	Volatile fatty acids (VFAs such as proprionic acid, butyric acid, acetic acid), Ethanol	Volatile fatty acids (VFAs such as proprionic acid, butyric acid, acetic acid), Methanol, Butanol, Acetone
Parameters affecting H_2_ yield	⁃ Light intensity ⁃ Medium composition ⁃ Microorganism type ⁃ Photobioreactor design	⁃ Light intensity ⁃ Medium composition ⁃ Microorganism type ⁃ Photobioreactor design	⁃ Medium composition ⁃ Microorganism type ⁃ Light intensity ⁃ Type of substrate ⁃ Photo-fermenter design	⁃ Type of substrate ⁃ Medium composition ⁃ Microorganism type ⁃ Fermenter design
Gases produced	⁃ H_2_, O_2_	⁃ H_2_, O_2_, CO_2_	⁃ H_2_, CO_2_	⁃ H_2_, CH_4_, CO_2_, CO, H_2_S
Advantages	⁃ Simple substrate: H_2_O ⁃ CO_2_ consumption ⁃ Simple condition for cultivation	⁃ Separate the requirements for O_2_ and H_2_ production. ⁃ Capable of fixing N_2_ from air (to generate nitrogenase) ⁃ Metabolite by-product converted to H_2_	⁃ Process broad range of substrates ⁃ Bioremediation ⁃ Process effluent from dark fermentation ⁃ Photosynthetic bacteria can use a broad spectrum of light	⁃ Process broad range of substrates ⁃ Bioremediation ⁃ Process independent of light ⁃ The created by-products bring value to the process ⁃ O_2_ restriction is of no concern
Opportunities	⁃ Genetic and metabolic engineering ⁃ Optimization of culture conditions, light usage efficiencies, and reactor design	⁃ Avoid H_2_ consumption by the hydrogenase enzyme. ⁃ Investment and operation cost reduction	⁃ Optimization of substrate usage, process, and reactor design ⁃ Genetic engineering ⁃ Cell immobilization	⁃ Genetic engineering ⁃ Optimization of substrate utilization, process, and reactor design
Considerations for commercialization	⁃ Demand high light intensity ⁃ Demand bioreactors with huge surface areas ⁃ Reduction of O_2_-sensitivity ⁃ Formation of explosive O_2_–H_2_ mixture ⁃ Low photochemical efficiency	⁃ Demand high light intensity ⁃ Demand bioreactors with huge surface areas ⁃ Elimination of uptake hydrogenase to avoid H_2_ decomposition	⁃ Substrate pre-treatment ⁃ Insufficient light conversion efficiencies ⁃ Inhibition of hydrogenase by O_2_	⁃ Substrate pre-treatment ⁃ Insufficient substrate conversion efficiency ⁃ Low H_2_ purity in gaseous product mixture ⁃ Relatively lower H_2_ yield

Bio-photolysis is characterised by low H_2_ yields (the photo-hydrogen-energy conversion efficiency is less than 10%) [136], due to inefficient light conversion and oxygen sensitivity of the process. Utilising O_2_-binding proteins allows for the control of O_2_ production during the photosynthesis process. Adding an inert gas to the reactor headspace can also assist in minimising O_2_ concentrations, however, this process entails a significant operational expenditure. One effective strategy for enhancing H_2_ production is through genetic and metabolic engineering of cyanobacteria and green algae to increase the light conversion efficiency [137,138]. Additionally, direct bio-photolysis might cause safety issues since the mixture of O_2_ and H_2_ could be explosive. Furthermore, this light-driven process produces hydrogen gas only when microalgae are exposed to light. While sunlight is a reasonably affordable energy source, the system might still need artificial illumination to further increase hydrogen production efficiency, thereby increasing the cost of bioreactors and increasing energy expenses [139]. Other parameters affecting biohydrogen output include microalgae light-capturing and CO_2_ fixation efficiency [140]. Many techniques for optimizing H_2_ generation via bio-photolysis have been investigated including; bioreactor construction, bioprospecting [141], genetic and metabolic engineering of microalgae [142], as well as optimizing culture and process parameters [143].

Further research to improve the maturity of biohydrogen production should focus on selecting appropriate microorganisms, pre-treatment of substrates, process and reactor parameter optimization, and H_2_ extraction from product gases. Lee et al. [154] suggested that dark fermentation is kinetically faster than photo-fermentation or bio-photolysis, however, the liquid by-products from dark fermentation such as lactic acid (C_3_H_6_O_3_), butyric acid (C_4_H_8_O_2_), acetic acid (CH_3_COOH), butanol (C_4_H_10_O), methanol (CH_3_OH), or acetone (C_3_H_6_O), limit the maximum efficiency of H_2_ generation. It was thus recommended to select and domesticate mixed cultures to reduce by-product generation and improve hydrogen formation rates. Singh and Wahid [155] claimed to use immobilized whole cell techniques in photo and dark fermentation to improve the efficiency of hydrogen production.

## 3 Cost and life cycle environmental impacts comparison

### 3.1 Cost analysis

#### 3.1.1 Cost of hydrogen from electrolysis

The costs of water electrolysis-based hydrogen production technologies can be categorized under capital expenditures (CAPEX, including the cost of electrolyser, liquid compressor, gas compressor, storage tank, electricial connection, heater, installation and indirect cost [156]) and operating expenditures (OPEX, including the cost of electricity, maintenance, labour, water and fixed operation & management [157]). The levelized cost of hydrogen (LCOH) is a parameter that can be utilized to compare the costs of different hydrogen production techniques. LCOH is defined as the ratio of the overall costs (including CAPEX and OPEX) throughout the full project duration to the total quantity of energy carrier produced at the same time [158]. The LCOH equation can be expressed as per Eq. (10), where t denotes the year number during the lifetime of the hydrogen production plant, C_t_ represents cost at “t”, Q_t_ is the amount of hydrogen produed at “t”, I_0_ refers to initial investment cost, and r represents discount rate.

(10) $$\text{LCOH}=\frac{I_{0}+\sum_{t=0}^{n}\frac{C_{t}}{{\left(1+r\right)}^{t}}}{\sum_{t=o}^{n}\frac{Q_{t}}{{\left(1+r\right)}^{t}}}$$

The cost of reliable zero-carbon electricity (such as from wind or solar energy) equates to roughly 50%–55% of the LCOH on average [51]. The capital cost of the electrolyser accounts for 15%–20% of the LCOH [159]. Financing and fixed operating costs (such as plant upkeep and maintenance) represent approximately 18%–24% of the LCOH [160]). The magnitude and variety of these costs have a significant impact on the LCOH when producing green hydrogen. In the existing literature on techno-economic modeling analysis of hydrogen production through water electrolysis, the LCOH ranges between 2.34 and 6.55 €_2021_/kg [156,161,162]for AE technology and 3.77–9.50 €_2021_/kg [[163], [164], [165]]for PEM technology. However, most of these ranges are based on theoretical mathematical modelling and do not account for complex factors encountered in practical engineering applications (such as taxes, the fluctuation of renewable electricity price, and regional costs of renewable energy acquisition). Therefore, the actual LCOH of hydrogen production in industrial applications is expected to be relatively higher than that of the theoretical estimates. Zhiyuan et al. [157] assessed LCOH values for different electrolysis technologies in different regions based on data from the IEA (International Energy Agency) and IRENA (International Renewable Energy Agency). They found that for a typical PEM electrolyser facility of 10 MW capacity in the EU, the average LCOH would be 11.06 €_2021_/kg and 11.61 €_2021_/kg for wind and solar scenarios respectively (a higher capacity factor is largely responsible for the relatively lower LCOH for the wind scenario than that of solar utility in the EU). Minutillo et al. [166]assessed the on-site hydrogen refueling stations using grid connected PV plants and electrolysis units in Italy. Their results indicated that LCOH from AE technology ranges from 9.29 to 12.48 €_2021_/kg. However, existing literature widely acknowledges that the cost of producing renewable electricity continues and will continue to decline with technology advancement and economies of scale [86,167,168], resulting in the LCOH to be declined to the range of 4.15–5.84 €_2030_/kg in 2030 [157].

**Fig. 4:**
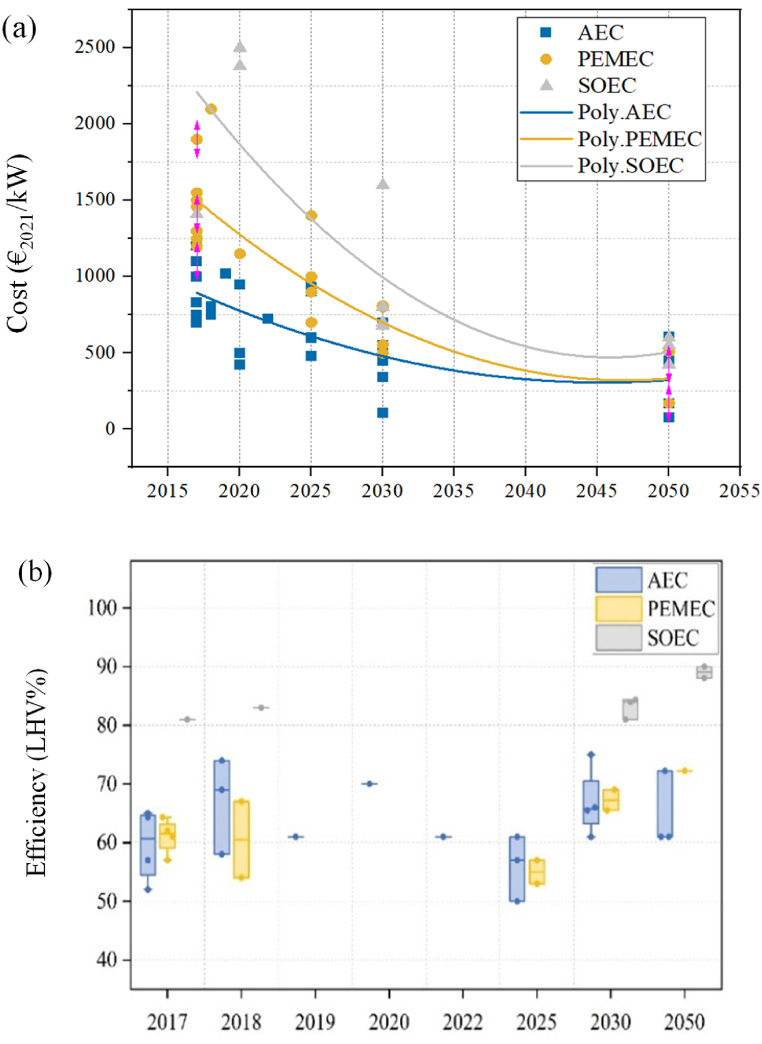
Cost reduction trend and efficiency of AEC, PEMEC, and SOEC to 2050. (a): cost reduction trend; (b) efficiency development (Data source: PIK) [60]. (AEC: alkaline electrolysis cell; PEMEC: polymer electrolyte membrane electrolysis cell; SOEC: solid oxide electrolysis cell; poly: polynomial fitting function).

For the three water electrolysis-based hydrogen production technologies, it is also anticipated that electrolyser CAPEX will decrease over time, lowering the cost of green hydrogen across all regions (especially for PEM and SOE) [169]. Glenk and Reichelstein [170] presented historical cost estimates up to 2016 and projections through to 2030. They projected yearly CAPEX savings of 3% for AE and 4.8% for PEM but did not predict the trend for SOE. Schmidt et al. [26] estimated the CAPEX of alkaline electrolysis cell (AEC), polymer electrolyte membrane electrolysis cell (PEMEC), and SOEC at 870, 1263, and 2854 €/kW in 2020, decreasing to 611, 978, and 1902 €/kW in 2030 respectively; this is higher than the estimate from Glenk and Reichelstein [170]. PIK (2021), on the other hand, conducted an updated CAPEX assessment of the cost and efficiency trend for AEC, PEMEC, and SOEC and projected the quantitative results to 2050 [60], as shown in Fig. 4.

#### 3.1.2 Cost of hydrogen from thermochemical cycles

There is on-going investigation into the direct use of nuclear heat for water molecule splitting using thermochemical cycles. The Cu–Cl and S–I cycles are currently the most promising low-temperature and high-temperature cycles respectively [171]. The LCOH of S–I and Cu–Cl cycles is suggested to lie between 6.72 and 10.15 €_2021_/kg H_2_ [[172], [173], [174]] and 5.54–7.58 €_2021_/kg H_2_ [175,176], respectively, with averages of 8.44 €_2021_/kg H_2_ and 6.56 €_2021_/kg H_2_. Due to the lack of large-scale industrial application of water thermochemical cycles, the LCOH values from literature are mostly based on software simulations and assume an ideal environment; such assessments must be seen as relatively indicative as they may not include for inefficiencies across the system such as heat loss and pressure drop in the transfer process [177]).

Plant capacity, as well as other parameters such as H_2_ production capacity, plant efficiency, and electricity cost, have a considerable impact on the cost of hydrogen, with published figures shifting significantly based on these input parameters even for the same type of energy source (such as nuclear or solar energy) and technology [178]. Cost data for the thermochemical cycle is limited, with few studies disclosing the capital cost data needed to calculate the LCOH. As a result, the LCOH estimations are subject to large and unquantifiable uncertainty. Currently the thermochemical cycles have only been deployed in pilot-scale testing by the Atomic Energy Agency of Japan [179] and further research and development by the Idaho National Laboratory [180]. Large-scale hydrogen production plants would be necessary to match the heat provided by nuclear facilities and to reduce the cost of hydrogen production.

#### 3.1.3 Cost of biomass-based hydrogen

Existing biomass gasification and biological conversion hydrogen production technologies do not have large-scale commercial applications, and therefore rely on system model simulations to calculate cost data. It is difficult to obtain normative parameters for techniques that are still in small-scale production. The inventory data that are utilized for the assessment either require additional self-defined parameters or are distinct from one another; this results in very disparate values for cost evaluation of biomass-based hydrogen production in the literature. Table 7 depicts estimations of hydrogen production cost from biomass gasification and biological conversion. Pallozzi et al. [181] investigated a 1 MWth input biomass gasification hydrogen production plant at a temperature of 573 K with a steam to biomass ratio of 2.0; the LCOH was estimated to be 8.3 €_2016_/kg (10.85 €_2021_/kg; Chemical Engineering Plant Cost Index (CEPCI) is used to update the LCOH value; CEPCI_2016_ = 541.7; CEPCI_2021_ = 708.0 [64]). Hamedani et al. [182] conducted a techno-economic analysis of a small-scale (100 kWth) hydrogen production system using biomass gasification and estimated the LCOH to be 12.75 €_2016_/kg (16.66 €_2021_/kg).

**Table 7:** Estimated costs of hydrogen production from biomass gasification and biological conversion.

Feedstock	H_2_ production method	Plant details	Capital expenditure	Operating expenditure	Revenue from H_2_	LCOH	Updated LCOH	Ref.
Food waste	Dark fermentation	Plant capacity - 2 ton/day; Lifetime - 10 years	931,020 €/a	299,746 €/a	639,920 €/a	14.91 €_2016_/kg	19.49 €_2021_/kg	[183]
Molasses	Dark fermentation	Plant capacity- 50 m^3^; Lifetime - 10 years	478,200 €/a	262,170 €/a	331,121 €/a	30.03 €_2016_/kg	39.25 €_2021_/kg	[184]
Food waste	Dark fermentation	plant capacity- 3 ton/day; Lifetime - 15 years	583,092 €/a	88,298.1 €/a	146,473.6 €/a	11.35 €_2016_/kg	14.83 €_2021_/kg	[185]
Wastewater	Dark fermentation	Plant capacity- 10 m^3^; Lifetime - 10 years	1,615,000 €/a	1,227,000 €/a	328,000 €/a	30.03 €_2012_/kg	36.37 €_2021_/kg	[135]
Agricultural waste	Dark fermentation	Plant capacity - 10m^3^; Lifetime - 10 years	2,097,000 €/a	1,238,000 €/a	328,000 €/a	30.03 €_2016_/kg	39.25 €_2021_/kg	[135]
Food waste	Dark fermentation	Plant capacity- 10 ton/day; Lifetime - 10 years	707,850 €/a	366,700 €/a	574,800 €/a	25.47 €_2016_/kg	33.29 €_2021_/kg	[186]
Lignocellulose biomass	Gasification	Biomass feeding rate - 20 kg/h; Lifetime −20 years	76,910 €/a	46,790 €/a	3700 €/a	12.75 €_2016_/kg	16.66 €_2021_/kg	[182]
Almond shell	Gasification	Biomass feeding rate - 20 kg/h; steam feeding rate – 20 kg/h	60,700 €/a	39,900 €/a	3625 €/a	10.37 €_2016_/kg	13.55 €_2021_/kg	[182]
Hazelnut shell	Gasification	Constant flow rate – 200 kg/h (1000 kW_th_) with a moisture content of 10%	1625.76 €/a	847.27 €/a	276.51 €/a	8.3 €_2016_/kg	10.85 €_2021_/kg	[181]
Nutshell	Gasification	Steam to biomass ratio – 1kg_steam_/kg_biomass_	603,420 €/a	420,904 €/a	372,482 €/a	15.7 €_2018_/kg	18.43 €_2021_/kg	[187]

(CEPCI: Chemical Engineering Plant Cost Index; CEPCI_2012_ = 584.6; CEPCI_2016_ = 541.7; CEPCI_2018_ = 603.1; CEPCI_2021_ = 708.0 [64]).

The LCOH of dark fermentation in literature is estimated to be between 14.83 and 39.25 €_2021_/kg [135,[183], [184], [185], [186]], while the LCOH range of biomass gasification is expected to be 10.85–18.43 €_2021_/kg [181,182,187]. The technological development of biomass-based hydrogen production remains highly uncertain until 2030, and therefore, its cost changes cannot be predicted with the same level of confidence as that for water electrolysis.

### 3.2 Life cycle environmental impact assessment

The utilization of Life Cycle Assessment (LCA) as an environmental impact assessment technique for products and services is well-established [188]. In accordance with ISO standards 14040 and 14044 [189,190], this methodology is structured into four distinct stages. Firstly, the “Goal and Scope Definition” phase is employed to establish the objective of the assessment and define the system boundaries. Secondly, the “Life Cycle Inventory Analysis” stage entails assuming and calculating all pertinent input and output parameters. Thirdly, the “Life Cycle Impact Assessment” stage is implemented to quantify the environmental consequences associated with the evaluated process chain. Lastly, the “Life Cycle Interpretation” phase is undertaken to deliberate upon the findings. A range of studies examing hydrogen production have been conducted using LCA methods. Delpierre et al. [191] used LCA methods to compare the environmental impacts of large-scale AE and PEM systems for CO_2_-free hydrogen production in the Netherlands. Their results show that both systems have similar environmental impacts, with the electrolyser contributing to only 10% of the total impact. The origin of electricity, even when derived from renewable sources, is the main contributor to environmental impact, emphasizing the need for clean energy sources in hydrogen production. Zhang et al. [192] conducted a comprehensive life cycle assessment (LCA) for three solar-based hydrogen production methods. Their results indicate that the thermochemical water splitting method using the S–I cycle coupled with solar photothermal technology exhibits low global warming potential (GWP) (1.02 kg CO_2_-eq/kg H_2_) and acidification potential (6.56E-3 kg SO_2_-eq/kg H_2_), demonstrating significant environmental advantages in the overall ecosystem impact. Bhandari et al. [193] conducted a LCA of hydrogen production via electrolysis and found 96% of GWP is associated with the set up of the turbine and H_2_ compression/storage in wind electrolysis. In the context of hydrogen production technologies of this paper, the system boundary is limited to the cradle-to-gate perspective, whereby the "gate" refers to the end of the hydrogen production unit [194].

**Fig. 5:**
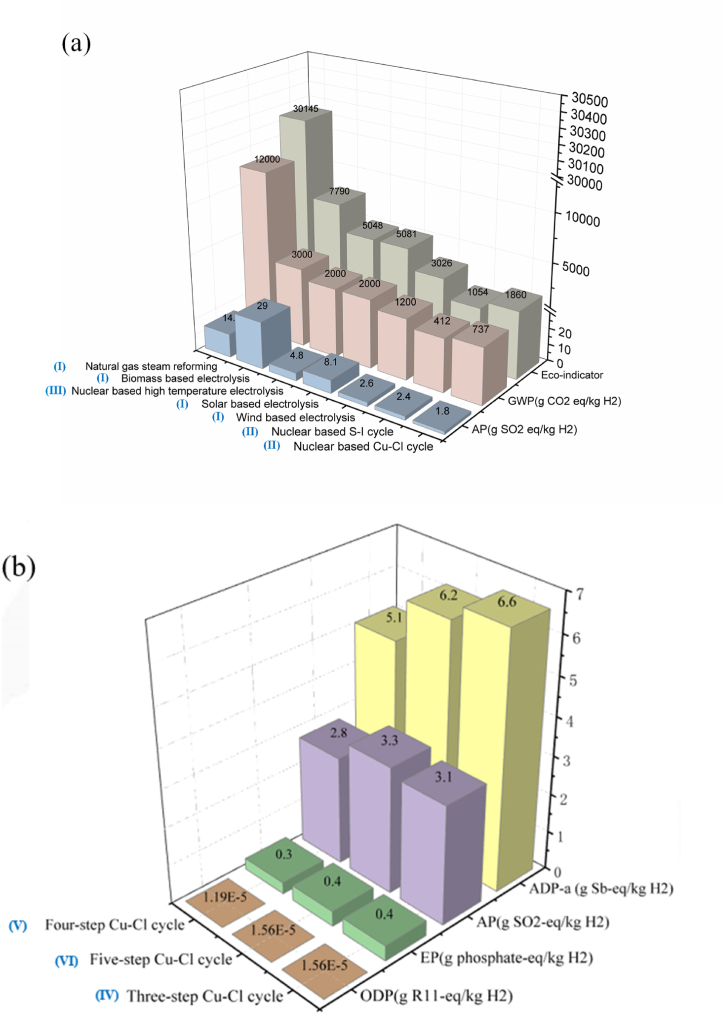
LCA analysis results of different sustainable hydrogen production technologies. (a) GWP, AP and Eco-indicator value; (b) ODP, EP, AP and ADP-a value [197] (AP: acidification potential; ADP-a: abiotic resource depletion potential; EP: eutrophication potential; GWP: global warming potential; ODP: ozone depletion potential). System assumptions: (Ⅰ) National Renewable Energy Laboratory provide GWP information for hydrogen produced by traditional natural gas steam reforming, wind and solar based electrolysis [196]. (Ⅱ) Plant capacity 3000 kg H_2_/day; overall inputs for 1 h of operation of hydrogen production plant (thermal energy 18.57 GJ; water 1125 kg); overall output for 1 h of operation of hydrogen production plant (hydrogen 1125 kg), heat exchanger efficiency (HHV) = 50% [198]. (Ⅲ) Nuclear based high temperature electrolysis plant requirement: electrical energy 200 MJ/kg H_2_, thermal energy 35 MJ/kg H_2_. Hydrogen production rate 7700 kg/h; plant capacity 600 MW [199]. (Ⅳ) Three-step Cu–Cl cycle hydrogen plant overall inputs: thermal energy 182.74 MJ/kg H_2_; electrical energy 67.15 MJ/kg H_2_; water 9 kg/kg H_2_. Plant outputs: oxygen 8 kg/kg H_2_; hydrogen 1 kg [197]. (Ⅴ) Four-step Cu–Cl cycle hydrogen plant overall inputs: thermal energy 161.05 MJ/kg H_2_; electrical energy 67.15 MJ/kg H_2_; water 9 kg/kg H_2_. Plant outputs: oxygen 8 kg/kg H_2_; hydrogen 1 kg [197]. (Ⅵ) Five-step Cu–Cl cycle hydrogen plant overall inputs: thermal energy 195.7 MJ/kg H_2_; electrical energy 50.3 MJ/kg H_2_; water 9 kg/kg H_2_. Plant outputs: oxygen 8 kg/kg H_2_; hydrogen 1 kg. Plant capacity for three Cu–Cl cycles: 125,000 kg H_2_/day. Plant lifetime: 60 years [197].

Natural gas steam reforming technology (for grey hydrogen) was employed as a control in previous studies for comparison to make the results of LCA analyses of different sustainable hydrogen production technologies more relative [195]. Fig. 5(a) depicts the GWP, acidification potential (AP), and eco-indicator (equivalent to 10 times the acidification potential value plus 2.5 times the GWP value) of seven hydrogen production techniques. The nuclear based S–I cycle showed the lowest GWP (412 g CO_2_-eq/kg H_2_; 3.4 g CO_2_/MJ), whereas natural gas steam reforming had the highest (12000 g CO_2_-eq/kg H_2_; 100 g CO_2_/MJ). In terms of AP, nuclear based Cu–Cl cycle displayed the lowest value (1.8 g SO_2_-eq/kg H_2_; 0.015 g SO_2_/MJ) while biomass-based electrolysis showed the highest value (29 g SO_2_-eq/kg H_2_; 0.242 g SO_2_/MJ) [196]. An eco-indicator was used to represent or assess the entire environmental impact by Ozbilen et al. [197]. When compared to steam reforming, the results revealed that producing hydrogen using renewable energy-based electrolysis and a nuclear-based thermochemical cycle had a substantially lower environmental impact.

It should be emphasised that the emissions from natural gas steam reforming or biomass-based hydrogen production are fundamentally different from those from water electrolysis technologies powered by nuclear, wind, or solar energy. The plant operation, which occurs continuously, is the main contributor to the emissions from natural gas steam reforming and biomass-based hydrogen production. Conversely for electrolysis-based technologies, mining, manufacturing, and construction phases make up a large portion of the emissions, while plant operation itself makes up a minor portion; these initial processes prior to hydrogen production will lead to an initial significant amount of emissions, followed by relatively lower emissions over the period of plant operation (20 or so years). In order to decrease the environmental impact, acid gas neutralisation and carbon dioxide sequestration technologies may be effective [198].

For the water thermochemical cycles, Ozbilen et al. [197] published a comparison of life cycle assessments for three-, four- and five-step Cu–Cl cycle designs. Fig. 5(b) depicts the results of their analysis in terms of abiotic resource depletion potential (ADP-a), AP, ozone depletion potential (ODP), and eutrophication potential (EP) of hydrogen production from three-, four-, and five-step Cu–Cl cycles based on nuclear energy. Because of the lower thermal energy required, the four-step Cu–Cl cycle has the lowest AP value (2.8 g SO_2_-eq/kg H_2_; 0.023 g SO_2_/MJ), whereas the three-step Cu–Cl cycle has the greatest AP value (3.3 g SO_2_-eq/kg H_2_; 0.028 g SO_2_/MJ). The four-step Cu–Cl cycle also has the lowest ADP-a (5.1 g Sb-eq/kg H_2_; 0.043 g Sb/MJ). The EP value indicates the effects of high macronutrient concentrations in the environment, and the values of three-, four- and five-step Cu–Cl cycles are similar (approximately 0.4 g Phosphate-eq/kg H_2_; 0.003 g Phosphate/MJ). ODP implies depletion of the stratospheric ozone layer because of emissions and increased ultraviolet radiation. Their results revealed that four-step Cu–Cl cycle has the lowest ODP values, while three-step and five-step Cu–Cl cycles are almost identical.

## 4 Conclusions

This paper compared the research status, technology readiness level, characteristics, and large-scale deployment barriers of several hydrogen production techniques. Polymer electrolyte membrane electrolysers have the advantages of high energy efficiency (58%–65%), high purity of generated hydrogen (99.999%), relatively short response time to rapid power changes (milliseconds), and the ability to combine variable renewable electricity producers. Optimistic scenarios suggest that the levelized cost of green hydrogen from polymer electrolyte membrane water electrolysis will be in the range of 4.15–6 €/kg in 2030, and thus is suitable for large-scale industrial application in the near term. The sulfur-iodine cycle and copper-chlorine cycle have the greatest potential for large-scale application among the thermochemical cycles. These two technologies are suitable for combination with nuclear energy to generate sustainable hydrogen at expected relatively low prices typically in the range 5.5–10.2 €_2021_/kg H_2_. However, it is difficult to predict future prices due to the lack of present commercial applications of the technology. Biomass gasification processes (steam gasification and supercritical water gasification) offer significant potential in geographic regions with widespread availability of woody crops (such as in Canada and Scandinavian countries) for the production of large quantities of syngas. As a relatively mature technology (TRL 8), large-scale biomass gasification industrialization can be achieved in the near term should this technology effectively solve the issue of tar formation and product gas separation. With the issue of cost unpredictability (the levelized cost of hydrogen via dark fermentation is suggested to be in the range 14.83–39.25 €_2021_/kg) and low system efficiency (less than 10% of direct biophotolysis), biological conversion technologies still need considerable development to be competitive with PEM water electrolysis technology.

## CRediT authorship contribution statement

**Yunfei Li:** Conceptualization, Formal analysis, Investigation, Methodology, Writing – original draft. **Richen Lin:** Conceptualization, Investigation, Methodology, Supervision, Validation, Writing – review & editing. **Richard O'Shea:** Conceptualization, Supervision, Writing – review & editing. **Vaishali Thaore:** Validation, Writing – review & editing. **David Wall:** Project administration, Supervision, Validation, Writing – review & editing. **Jerry D. Murphy:** Conceptualization, Funding acquisition, Project administration, Supervision, Validation, Writing – review & editing.

## Declaration of competing interest

The authors declare that they have no known competing financial interests or personal relationships that could have appeared to influence the work reported in this paper.
